# The Eco‐Friendly Preparation of Se, Zn, and Ag MONPs and Their Current Medical Applications and Drug Delivery for AD Diseases

**DOI:** 10.1155/tswj/7213377

**Published:** 2026-07-02

**Authors:** Wessal M. Khamis, Hanaa Hassan Hussein, Youssra Kareem Jaber Al Hilaly

**Affiliations:** ^1^ Mustansiriyah University, College of Science, Department of Chemistry, Baghdad, Iraq, uomustansiriyah.edu.iq

**Keywords:** drug delivery, medical application, MONPs, nanoparticle fabrication, nanotechnology

## Abstract

In recent decades, significant progress has been made in the field of nanotechnology that enabled researchers to promote and create nanoshaped compounds with attractive physicochemical properties. These materials were used in different biomedical applications, because of the superior quality of nanoparticles and nano‐oxide materials compared with their bulky counterparts in the fields of medicine and healthcare. This gives a push to this line of science toward making a great progress. Despite the positive benefits associated with nanofield usages, nanomaterials have cytotoxic effects which are directly associated with their physiochemical properties. Therefore, proper control of factors governing nanoparticle synthesis can improve biocompatibility and minimize toxicity, which would enable researchers to achieve more suitable outcomes. In our review, we focus on the significant progress in the field of MONPs, covering their synthesis and applications, as well as the methods of preparations and their chemical or physical qualities. In addition to their antibacterial effectiveness, MONPs also exhibit biological effects, like antitumor, anticancer, proregenerative, and drug delivery properties. The last part of this review highlights the use of oxide nanoparticles for drug delivery in brain illness therapy and their potential in the treatment of brain diseases.

## 1. Introduction

Nanotechnology, over the past decades, has become an important field of science, attracting interests from scientists [1]. This field has gained significant importance because of the wide clinical applications of metal oxide nanoparticles (MONPs), whose properties depend on their nanosize, which leads to a high surface area and is influenced by the suitable preparation method used [2, 3]. The nanosize with a high surface area enabled new methods for characterization and treating diseases, which were previously inaccessible due to the size limitation of conventional materials. These materials offer various benefits, including high stability. Their ease of preparation allows good control over aspects such as size, shape, porosity, and other desirable properties. All these properties enable nanoparticles to penetrate cells effectively. MONPs have attracted significant attention in the field of drug and healthcare industries [4]. Through progress in the design and engineering of MONPs, the limitations associated with their large counterparts can be at last overcome, allowing scientists to achieve astounding breakthroughs in applications like targeted drug delivery, bio‐imaging, and sensors, where MONPs act as biomolecules, etc. [5, 6]. To take an example, Kilic and his coworkers have been interested to study the ecofriendly synthesis of MONPs. They have also reviewed their medical applications as therapeutic agents or drug delivery systems, as well as their in vivo and in vitro biomedical applications for various MONPs [7]. Moreover, due to their nanosize, MONPs have the ability to interact more deeply with different cellular structures compared with their bulk counterparts. More importantly, they have reduced toxicity toward cellular components because of their high biocompatibility [5, 6]. Presently, numerous types of MONPs have been utilized in clinical research as antibacterial agents, in addition to wound healing dressings, biosensors, anticancer therapies, and image contrast agents [7]. Among these nanomaterials, zinc oxide nanoparticles (ZnO NPs), along with silver oxide nanoparticles (AgO NPs) are important candidates for clinical and medical applications. This is backed up by research information obtained over the past 10 years regarding their in vivo and in vitro biological effectiveness. ZnO nanostructures exhibit low toxicity and are widely regarded as biocompatible biocompounds with remarkable properties that depend on their size, shape, orientation, and morphology, as well as their height and width [8]. MONPs have been widely utilized in traditional applications, such as sunscreens, ointments, food packaging, and cosmetic and personal care materials. Moreover, ZnO nanoparticles have antibacterial activity against microorganisms, basically due to their distinct properties, influenced by dose, time, and synthesis procedure [8]. Besides, because of their ingrained anticancer effects, ZnO NPs have received agreement and approval from the Food and Drug Administration (FDA) as a novel and efficient antitumor treatment [9]. Generally, it may be appropriate in that, besides the generation of high levels of reactive oxygen species (ROS), ZnO NPs may activate cellular sites and exert cytotoxic action toward cancer cells by modulating the equilibrium of zinc‐dependent protein activity [10]. However, ZnO NPs have the ability to induce toxic influences in various cells of humans or organisms, thereby further studies are necessary. Nevertheless, ZnO NPs have been shown to induce toxic outcomes in different cell types across various organisms, requiring supplemental studies to address their therapeutic potential while considering their prospective toxicological prohibitions [11].

**Figure 1:**
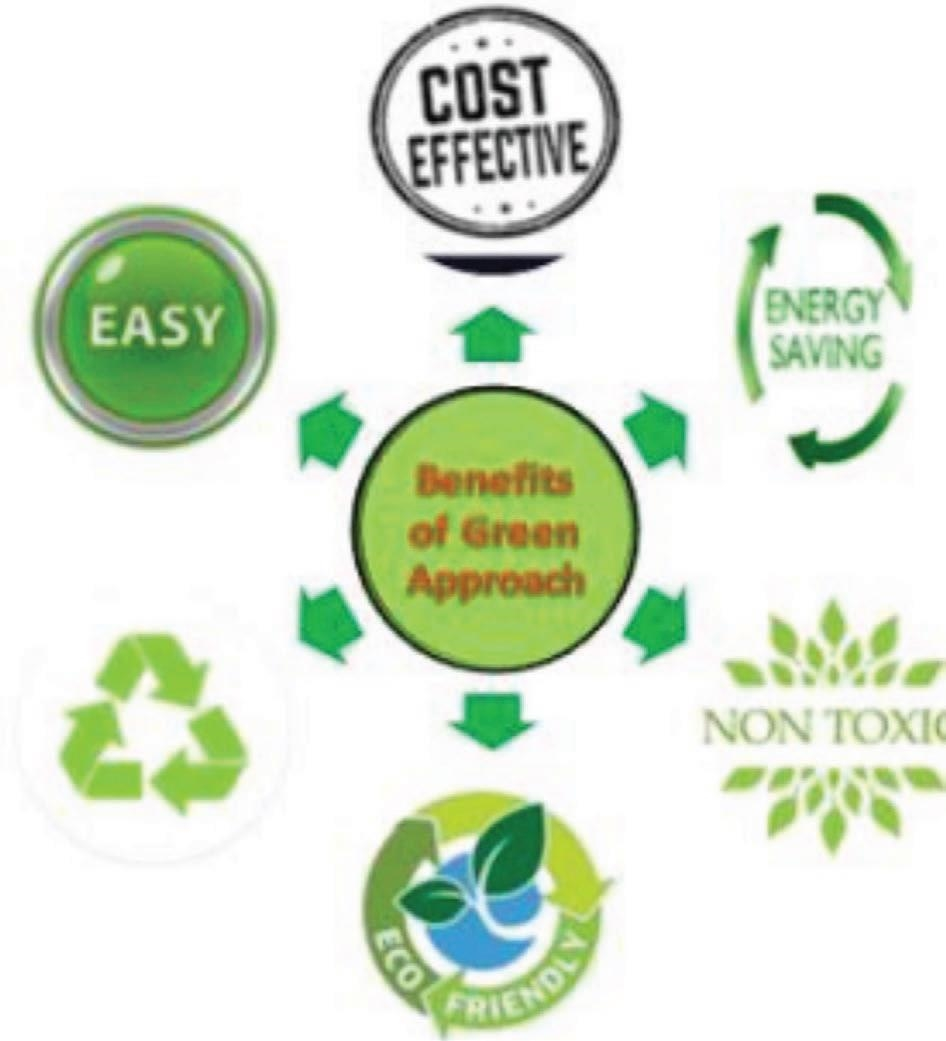
The key properties of eco‐benign nanoparticles [15].

Goktas et al. prepared ZnO, ZnO:Co (5%) nano rod‐like thin films, and ZnO/ZnO:Co (5%) homojunctions to evaluate the photocatalytic activity of these MONPs [12]. Silver oxide and other silver compounds have been used for therapeutic and antibacterial applications for thousands of years [11, 12]. The ancient civilizations utilized silver in making eating utensils and vessels to store water, food, or wine to prevent spoilage. Silver compounds were used in the past for ulcer treatment and wound healing. Also, silver nitrate has been used for the treatment of wound and disinfection of medical tools. Silver synthesis was promoted for the treatment of wound infections and burn care [13]. According to the principles of green chemistry, phytochemicals derived from biomolecules can act as reducing and capping/stabilizing agents throughout the synthesis process. In recent years, plant‐mediated synthesis of nanomaterials has attracted significant attention due to their rapidity. The eco‐benign preparation of NPs is a method consistent with the principles of green chemistry, in which phytochemicals discharged from biomolecules act as both reducing and capping/stabilizing agents throughout the experiment. Over the past few years, plant‐mediated production of nanoparticles has gained considerable attention due to their rapidity and simplicity [14] (Figure 1). In nanostructure synthesis using plant extracts, plant leaves are typically mixed with wet sample of Ag salt sol at 25°C; therefore, these methods are considered part of the green chemistry field [15, 16]. Silver ions will be reduced from Ag metal salt initially by using reducing agents. Then, these reduced silver atoms will agglomerate to form tiny clusters, which eventually develop into Ag_2_O nanoparticles [17]. Ag_2_O nanoparticles were prepared by Ravichandran et al. by biosynthesis using *Callistemon lanceolatus* leaf extract [18]. They reported that the leaf extract produced hexagonal Ag_2_O nanoparticles with sizes around 3–30 nm. In another study, *Centella asiatica* was utilized to synthesize Ag_2_O nanoparticles at 25°C [19]. In the same way, Yu et al. used extracts of *Lippia citriodora* to synthesize Ag_2_O nanoparticles [19]. Ag_2_O nanoparticles were prepared in this way and simultaneously biosynthesized using leaf extract of *Artocarpus heterophyllus*. These A_2_O nanoparticles have a spherical shape with an average size of 14 nm. Finally, Sharma et al. discovered that flavonoids found in the leaves were responsible for the successful synthesis of Ag_2_O nanoparticles [20]. Some plants have been investigated for the biomimetic production of Ag_2_O nanoparticles. H. Abdullah et al. prepared a novel type of polymer that was utilized as a matrix. Ag_2_O nanoparticles were synthesized using an environmentally green method, utilizing silver nitrate as the substrate and sodium hydroxide as the precipitation compound, in the presence of orange leaf extract as the reducing agent. Lastly, composites of AgO NPs with polymer matrices were prepared using a solution method. The resulting compound was calcined at 550°C, which is the calcination temperature for Ag_2_O [21]. There are few papers or studies that focus on SeO_2_ nanoparticles, but there are also many reports that highlight their preparation and application in our life or in the science field. Alghunaim prepared nanocomposite samples that consist of chitosan (Cs)/polyacrylamide mixture combined with various amounts of selenium dioxide nanoparticles that were synthesized and characterized. The deposition of SeO_2_ nanoparticles onto chitosan resulted in enhanced physical qualities [22]. This study concerns the association of selenium dioxide with a synthetic carboxymethyl cellulose/polyvinylpyrrolidone. These nanoparticles with CMC/PVP were classified as novel nanocomposite films prepared using a sol infusion technique. These prepared film compounds have been characterized and investigated using different techniques that confirmed the association between the nanoparticles and CMC/PVP compound. The prepared nanocomposite films affirmed that the molecular chain piece dynamics and dielectric qualities have been improved with the increasing amount of doped SeO_2_. These improvements in physical qualities (e.g., thermal, structural, optical, and dielectric) have been very useful and important for device implementations [23].

## 2. Methods of Nanoparticle Preparation

### 2.1. Top–Down Approaches

It refers to the process where larger or bulk compounds are cracked down into smaller particles known as the nanoparticles using various mechanical, chemical, or physical methods [24, 25].

a. Laser ablation: A process of pulsed‐laser‐induced quenching of fluid–solid interactions and pulsation laser evaporation, which can produce composite films that were sequentially deposited with thickness. Researchers have provided a theoretical framework for developing new methods and approaches used to synthesize nanomaterials. Laser irradiation in a fluid medium, also known as laser ablation in liquid (LAL), can significantly promote ablation efficacy. Bleb motion can be gained through water inflow and heat transport (conduction or convection), which aids in the removal of the ablation molecules that may redeposit on the target surface and limits oxidation. In addition, the fluid medium effectively cools the target materials, blocking imprudent heat amassing [26]. In addition, the fluid medium potently chills the targeting martials, preventing excessive heat accumulation. So, LAL is widely applied in laser cleansing as well as in laser shock peening. In recent decades, the use of fluid media leads to the improvement of surface quality and ablation efficacy of fine metals during laser processing, like Au and Ag. Therefore, this part focuses on the principles and applications of liquid‐assisted laser ablation, and the impact of NP size and shape [26].
b. Electrochemical etching: Penetrable silicon can be formed utilizing electrochemical etching or anodization in hydrofluoric sol. In this process, porous silicon is not obtained just by submersion in a solution of HF. Instead, an electric current is applied between two electrodes, silicon being the anode and platinum being the cathode in the HF sol, and this leads to formation of pores in the silicon layer. Electrochemical etching is a very simple and cost‐effective method in terms of apparatus and chemical used. As a rule, silicon is placed as the anode whereas platinum is used as the cathode. However, other metals may still be used as the cathode. A Teflon cell is commonly utilized due to its resistance to acid in contrast to a glass cell. The simplicity of this setup makes it easy and simple to modify. However, the pores are not uniform on both sides of the silicon because of the inhomogeneity arising from a decrease in lateral potentiality [27].
c. Mechanical milling/ball milling: Milling of chemical compounds has been widely used in mineral, ceramic, and powder industries. The goals of mechanical milling are particle size reduction, melding or mixing up, particle configuration alteration, and creation of nanoparticles. The most commonly used mills for these purposes are high‐energy ball mills, such as tumbler globule mills, vibratory mills, stellate mills, and attritor mills. An itemized description of the various mills for MM can be found in Suryanarayana′s review; however, a brief overview of high‐energy power globule mills is also presented [28].

A. Bottom‐Up Approaches

Nano‐atoms and minimolecules are assembled using bottom‐up approaches to form nanoshaped particles. These approaches include chemical and biological methods [29].

B. Chemical Routes:
  a. Chemical vapor deposition (CVD): By using a chemical process that involves the vapor phase precursors, thin coatings can be synthesized on the surface of the raw materials through CVD [30]. Precursors have been deemed suitable for CVD whether they manifest adequate volatility, high chemical purity, strong evaporation firmness, low cost, low toxicity, and long shelf life. Also, its decomposition should not produce any contaminants. Vapor phase epitaxy, metal‐organic CVD, atomic layer epitaxy, and plasma‐enhanced several variations of CVD. The advantages of these methods include the production of highly pure, dense, homogeneous, and durable nanomaterials [31]. CVD is an effective technique for synthesizing high‐quality nanocompounds [32]. It is also widely known for creating two‐dimensional nanomaterials [33].
  b. Sol–gel process: The sol–gel process is a wet‐chemical method that is commonly utilized to create nanocompounds. Metal alkoxides or other metal precursors in a sol are hydrolyzed and then thermally decomposed [34].

## 3. Hydrothermal/Solvothermal

This method involves the synthesis of nanoparticle materials. Hydrothermal creation utilizes a broad temperature range extending from ambient temperature to considerably high temperatures. Comparing this strategy with physical or biological approaches, the hydrothermal method offers a number of benefits. At higher temperatures, nanomaterials produced by means of hydrothermal method may become unsteady [35].

## 4. Coprecipitation

This is a solvent displacement method and also a wet‐chemical process. Solvents like ethanol, acetone, and hexane are classified as nonpolymeric dissolvent. The polymer phases may be either synthetic or natural. During mixing, prompt diffusion of the polymer‐sol into the non‐sol phase of the polymer occurs. Interfacial exertion between the two phases leads to the conformation of nanoparticles compounds [36]. This method had the natural capacity to output a high yield of nanoparticles dispersed in water, which may be one of its key advantages. This process is used to synthesize numerous traditional iron oxide NP‐based MRI contraindication agents [37].

## 5. Inert Gas Condensation/Molecular Condensation

Metal NPs can be utilized using this method in a large amount. Fabrication of NPs utilized the universal ineffectual gas compression method, which forms NPs by causing a metallic initiator to lapse in a passive gas atmosphere. For an obtainable temperature, metals vaporize at a high rapidity. Cu metal nanoparticles are synthesized via steaming copper element in a repository with argon, helium, and neon. The atom speedily loses its power via evaporative cooling in a dilute gas after being released by boiling. Liquid nitrogen has been utilized for cooling all the gases, facilitating the formation of nanoparticles ranging from 2 to 100 nm [38]. Medical applications of MONPs will be discussed in the next sections.

## 6. Blood–Brain Barrier (BBB)

**Figure 2:**
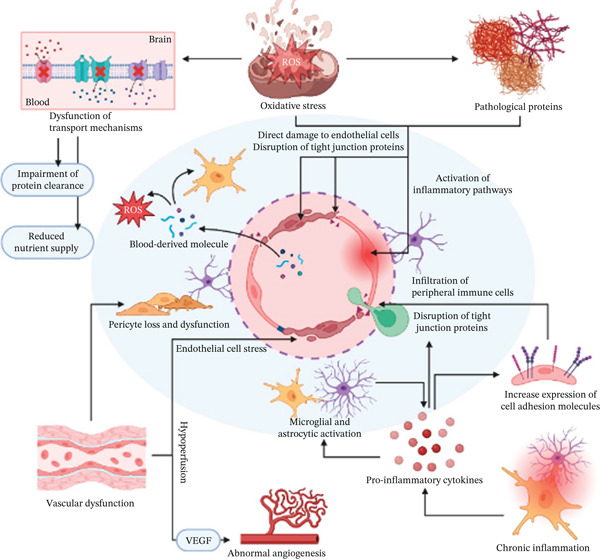
The mechanism of BBB disruption in the brain [46].

**Figure 3:**
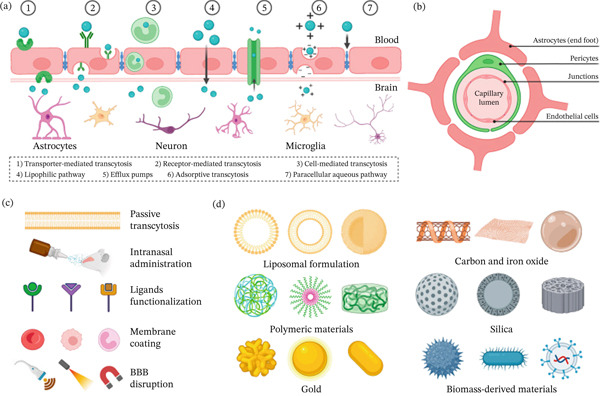
The strategies and materials for BBB regulation and brain‐targeted drug delivery. (a) Schematic diagram of different mechanisms for BBB crossing. (b) Schematic diagram of BBB structure. (c) Materials for brain‐targeted drug delivery. (d) Various noninvasive strategies for BBB crossing [53].

The human central nervous system (CNS) has built different kinds of barriers to save and defend itself from external influences like circulation of blood cell, chemical neurotoxins, and pathogen invasion [39, 40]. The structures′ permeability, which include BBB, blood–cerebrospinal fluid (CSF) barrier, blood–spinal cord barrier, and blood–retinal barrier, is of significant importance [41]. The blood vessels in the brain act as the BBB which itself is a very good defense system that was operated in a special structural organization [42]. The BBB consists of a thin layer of nonfenestrated endothelial cells enclosed by sleek muscle cells, pericytes, and astrocytic projections [43]. The major function of the BBB is to permit the transfer of ions, chemicals, and other cells between blood stream and the central nerve system. The protective barrier plays a crucial role in acting as a shield from potential deterioration of the human brain at common circumstances [44, 45]. Figure 2 showed the mechanism of BBB disruption at the brain. Disruption for the BBB has been a hallmark of some neurodegenerative diseases [46]. Therefore, this exceptional selectivity presents a huge hurdle when it comes to delivering remedies for the medical treatment of varying neurological diseases. Overcoming this hurdle demands medication to be active [47]. BBB can be defined as a semipermeable membrane which is located at the interface between the blood and cerebral tissue [48]. It is composed of endothelial cells for the capillary dike and pericytes entrenched in the capillary basement strata, tense segment, and astrocyte terminus‐feet that have been overlaying the capillary [49]. Pericytes closely envelope around 20% of endothelial cells. They regulate capillary blood flow of the brain through contraction and relaxation and act as a restraint for the divergence of vasoactive stimuli. Astrocytes refer to endothelial cells for BBB that have been ringed via astrocyte cell protrusions which provide biochemical support to those cells. Basal membrane is composed of laminin, Type IV collagen, proteoglycans, heparan sulphate, fibronectin, and other extracellular matrix proteins, where endothelial cells and pericytes are embedded. In pathological conditions, the plunge of the present extracellular matrix is directly associated to increased BBB permeability [50]. The BBB is formed by eclectic dense crosses between the endothelial cells that restrict solute motion. These tight junctions, which are made of tiny subunits of transmembrane proteins, recurrently connect endothelial cells at the interface between the blood and the brain, preventing paracellular permeability [51, 52]. Figure 3 represents the diagram that refers to the receptors mechanism for the medication crossing through in the BBB [53].

## 7. Anti‐Alzheimer Medications and Their Mechanisms of Action

**Figure 4:**
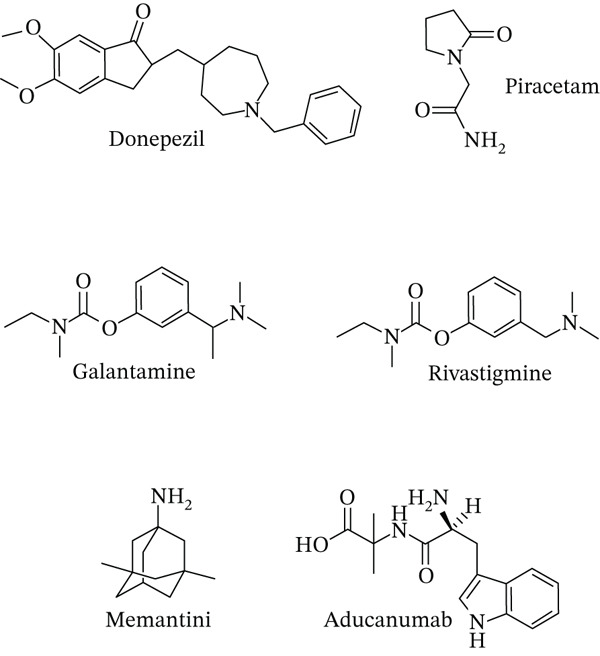
Chemical structure of anti‐Alzheimer drugs [57].

The US FDA has approved many types of medications for treating AD, renowned for their leverage and safety in managing signs. Those drugs consist of donepezil, rivastigmine (RHT), galantamine (as cholinesterase inhibitors), memantine (which acts as an NMDA receptor antagonist), aducanumab, and lecanemab (takes action as anti‐amyloid monoclonal antibodies) [54]. Acetylcholinesterase inhibitors (AChEIs) that have been used to treat Alzheimer′s disease (AD) are RHT, donepezil, and galantamine. AChEIs reduce ACh degradation in the brain of patients infected with AD, decreasing the activity of the enzyme acetylcholinesterase in the synaptic cleft [55]. In addition, they enhance central cholinergic neurotransmission and help slow the decline in cognitive functions. AChEIs therapeutics is commonly initiated after the diagnosis. AChEIs like RHT and donepezil are administered to treat mild, moderate, and severe cases of AD. Galantamine is mostly used to treat mild and moderate AD [56]. Noncompetitive, low‐affinity open‐channel blockers of the N‐methyl‐D‐aspartate receptor like memantine influence glutamatergic transmission and exhibited efficacy for curing moderate to severe AD [57]. Figure 4 shows the chemical structures of drugs used for treating AD.

Memantine has been a clinically effective therapeutic agent for treating different neurological disturbances, such as AD [58]. However, when compared with treatment and monotherapy, it has shown to be related with adverse effects like drowsiness. Generally, it is well tolerated, and its benignity has been contrasted with the placebo [59]. Researchers had proven that memantine is very effective as an uncompetitive antagonist of N‐methyl‐D‐aspartate receptor. Also, it is widely known for its moderate affinity [60]. Memantine significantly improved cognitive function and has the ability to address behavioral disturbances, outperforming placebo when used either as a monotherapy or in combination with donepezil [61]. Memantine doubtlessly works as an uncompetitive antagonist of NMDA receptors, a subtype of glutamate receptors in the CNS [62]. It reduces excessive stimulation [63]. It furthermore promotes antagonist action on nicotinic acetylcholine receptors and serotonin Type 3 (5‐HT3) [63]. Donepezil is marketed under the trademark Adlarity, which evidences a dependable transdermal delivery system that is applied weekly to the back, buttocks, or leg [64]. Donepezil is available in 5 and 10 mg/day oral formulation [65]. According to Larkin et al., this system has been promoted to constantly deliver remedy through the skin while preserving steady levels for effective therapy [66]. This manner of delivery may reduce the incidence of reciprocal gastrointestinal effects, associated with getting oral dosage of donepezil, resulting in improved cognitive outcomes patients with AD [67]. Its use at lower dosage is particularly suitable for sick people who have poor tolerability to standard therapeutic doses and for those who are at risk of developing cholinergic adverse effects, such as nausea, dizziness, anorexia, and vomiting [68]. Donepezil binds with cholinesterase enzymes and has reversible ability to inhibit action on them, especially acetylcholinesterase. This mechanism increases AChE levels at cholinergic synapses by preventing enzyme hydrolysis [69]. Galantamine is another type of AChEI that is well known for its ability to slow cognitive decline [70]. It is utilized to treat early‐stage AD and memory impairment. However, its efficacy is minimal for preceding juncture of dementia. The effects of these remedies focused on increasing the availability of acetylcholine. In addition, it inhibits the action of acetylcholinesterase by rupturing acetylcholine. It has been first sold under the trade name Nivalin at Bulgaria and other Eastern European states. Its reported adverse effects include muscle failure, muscular dystrophy, trigeminal neuralgia, and peripheral nerve pain [71]. Galantamine has been unparalleled as it acts like a potent allosteric potentiator for the *α*4*β*2 and presynaptic *α*7 nicotinic acetylcholine receptors. The mechanism safely reinforces emission of the enzyme acetylcholine at presynaptic neurons, underscoring clinical significance of its dual process of action [71]. Aducanumab is a fully human IgG1 monoclonal antibody which picks out a modulational epitope of *β*‐amyloid, a protein correlated with AD. This targeted manner has contributed to advancing our comprehension and treatment of this complex neurodegenerative state [72]. It was approved for the treatment of AD in June 2021 by the FDA [73]. Aducanumab effectually attaches to amyloid accumulations in both oligomeric and fibrillar forms, elucidating a distinct preference over amyloid monomers [74]. Strong amino acid interactions facilitate cursory and consolidated binding, outstandingly reducing interactions limited by the confined availability of epitopes within the monomers. In contrast, the potent affinity increases at higher concentrations for specified epitopes of the monoclonal antibody, leading to enhanced binding affinity [75]. RHT has been an effective cholinesterase inhibitor utilized in the treatment of mild to moderate AD, administered orally. Its effectiveness and the clinical use make it as a good choice for managing patients in these stages of the disease [76]. Oral dosage, which is commonly used, is associated with slower intake rate and considerable systemic aspect effects [77]. Compared with placebo, improved outcomes have been observed in the range of cognitive decline and activities of daily living; however, information are limited and clinical significance remains uncertain [78]. Moreover, RHT evidently contributed in the clinical understanding of treatment outcomes [79]. RHT effectually prohibits both acetylcholinesterase and butyrylcholinesterase via covalent linkage to the active sites of these enzymes, hence inhibiting their activity [80]. It stunts the hydrolytic action by AChE and BChE through interaction with catalytic sites. This drug has been efficient in curing dementia linked with Alzheimer′s and Parkinson′s diseases [81].

## 8. Nanostructured Lipid Carriers (NLCs)

TheNLCs are effective carrier systems consisting of an interior robust lipid matrix combined with liquid lipids [82]. They exhibit an effective carrying capability and stability [83]. These compounds have been utilized to deliver medicine from the nose to the brain and to treat AD associated with microglial activation [83]. NLCs offer some advantages, including simple manufacturing processes, lower levels of toxicity, and physical resistance. They permit drug release in response to the physiological demand, alongside high drug‐loading capacity. Moreover, NLCs prohibit drug percolation during storage while improving drug solubility and stability. These properties provide important merits over other drug delivery systems [84]. Their lipophilic nature and tiny particle/droplet size help them target the brain effectively [85]. This allows the drug to cross through the BBB, whereas its encapsulation within the lipid matrix protects it from enzymatic degradation. The barrier restricts the drug from reaching the brain at curative levels [86]. Scientist carried out a paired optimization of a formularization utilizing RHT‐loaded NLCs for instantaneous intranasal drug delivery. They harness a Quality by Design (QbD) approach, where the QTPP was established as a key requirement for efficient nose‐to‐brain delivery [87]. The in vitro drug profile showed a release of RHT from CS‐NLC that has been linked with drug propagation from the nanocarrier′s surface, followed by a prolonged remedy over time. Those findings evidenced that those CS‐NLCs can present prompt curative effectiveness, which may be of assistance for AD by promoting speedy drug release while constantly sustaining remedial activity after. The optimized formularization of RHT‐loaded NLCs, which developed out of high‐pressure homogenization (HPH), demonstrated remarkable steadiness and represents auspicious strategy for the efficient delivery of RHT directly to the brain via the nasal route. Their lipophilic nature and tiny particle/droplet size make them efficacious for targeting the brain [88]. Researchers have also reported the development of an unprecedented in situ clotting process. In this method, RV‐NLCs containing 6.25% (w/w) resveratrol (RV) has progressed during the formularization process. The obtained information exhibited a sustained release of IN and IV NLCs in comparison with RV solutions administered through similar routes. Moreover, the NLCs showed a rise in AUC and T1/2 values [89]. Scientists have also developed NLCs loaded with curcumin and donepezil particularly for brain drug delivery system through the nasal route. The NLCs exhibited a particle size below 50 nm, making them extremely efficacious for nasal delivery. In vivo pharmacokinetic studies clearly presented that this approach accomplished a notable outcome; the drug exhibited a notably higher concentration in the brain when administered via this route compared with the intravenous path [90]. Scientists also stated a perfect formularization of chitosan‐covered NLCs to improve brain delivery efficacy after intranasal administration. Studies on the allocation of CS‐NLCs which have been labeled with a near‐infrared dye distinctly illustrated that these nanocompounds could be delivered to the human brain through inhalation. This efficiency has been attributed to chitosan′s vigorous mucoadhesive properties, which reduce mucociliary clearance and allow the drug to reach the olfactory region successfully [91].

## 9. Polymeric Nanoparticles

Polymers are macromolecules composed of numerous recurring units which are arranged in a chain‐like molecular structure and have a wide range of compositions, formation, and effects. They have been utilized to develop nanoparticle systems optimized for particular biomedical applications because of their diverse compositions, structures, and advantages. Polymeric nanomaterials have been essentially harnessed in drug delivery, bioimaging, and biosensing applications [92]. The application of nanostructured materials in drug delivery has gained significant attention caused by the growing prominence of targeted drug delivery systems, which is why much effort has been devoted into developing polymeric nanoparticles that have systematic, tissue‐specific, and safe properties. Several effective methods exist for the use of nanomaterials in drug delivery, depending on the process utilized to linked the drug to the nanoparticles. Nanoparticle compounds may include polymeric nanocomposition or polymeric nanostructured conjugates, amphiphilic marrow/rind‐shaped assemblies, similar to polymeric micelles structures, or hyperbranched macromolecules with nanometer dimensions, commonly referred to as dendrimers [93]. The main limitations include particle aggregation, polymer chemical stiffness, raw materials used for the fabrication of nanoparticles, as well as the timely release of the buoyant substance, critical to guarantee optimal outturns [94]. Polymeric nanoparticles can dominate the drug release either through circulation within the polymer matrix or through matrix degradation. Research has focused on their application as drug delivery systems to enable site‐specific targeting of growths and to expedite the transport of pharmaceuticals across biological barriers, particularly the BBB [95]. Researchers have developed a strategy to synthesize efficient transporters of siRNA, a type of genetic substrate, for brain‐targeted delivery [96]. The nanocomponents (CT/siRNA) were composed of CGN‐ PEGylated poly(2‐(*N, N*‐dimethylamino) ethyl methacrylate) (PEG‐PDMAEMA) and Tet1‐PEG‐PDMAEMA in a weight ratio of 1:1. The outcome showed a successful synthesis of a targeted gene delivery method for treating AD by means of targeting brain neurons. Biodistribution and pharmacokinetic studies showed that the half‐lives of CT/Cy3‐siRNA, C/Cy3‐siRNA, and M/Cy3‐siRNA in the blood were 0.97, 1.00, and 1.01 h, respectively, which were significantly protracted compared with that of pristine Cy3‐siRNA (only 0.15 h). The extended blood circulation of PEGylated nanocomponents has been salutary for the delivery of siRNA to the brain. The maximum concentration of CT/siRNA and C/siRNA was 2.42 ± 0.18%ID/g‐brain and 2.21 ± 0.28% ID/g‐brain, respectively, which were distinctly higher than that of naked siRNA (0.11 ± 0.03%ID/g‐brain) and M/siRNA (1.05 ± 0.18%ID/g‐brain). The nanocomponents effectively traversed the BBB and successfully transmitted siRNA to neurons. The findings revealed that CT/siRNA nanocomponents have considerable potential as a cure AD [97]. According to scientists, PLGA nanospheres loaded with curcumin exhibited a strong inhibitory effect on A*β* aggregation, making them an auspicious drug delivery strategy for the treatment of AD [98]. Researchers had elucidated that TQ‐containing PLGA‐NPs together with polysorbate‐80 (P‐80) can safely and effectively facilitate the delivery of NPs across BBB into the brain [99]. Scientist have developed selenium NPs incorporated into poly lactic‐co‐glycolic acid (PLGA) nanoparticles, which have spherical carriers that were used to load curcumin. The outcomes exhibited vigorous inhibitory effects on A*β* aggregation in a transgenic mouse model of AD, underscoring a promising drug delivery method for Alzheimer′s therapy [100].

## 10. Metal Nanostructured

Metal nanocompounds have been suggested to be both carriers and therapeutic agents in the biomedical field because of their typical physicochemical outcomes and properties. They are also widely utilized in a variety of biomedical field. The metal nanoparticle components may be devised with dissimilar ligands to control their size and formation, allowing them to be utilized in drug delivery, diagnostics, and the treatment of the CNS diseases [101]. Noble metal‐based nanoparticles that have been used in medicinal applications exhibit higher biocompatibility and reliability, as well as the potential for large‐scale production without using organic compound solvents, consequently offering favorable interactions with biological frameworks [102]. The efficacy of nanoparticle‐based brain‐targeting systems depends upon critical factors, such as particle size for the nanometric scale, surface charge, and morphology. However, the most crucial factor has been the molecular assessment and interactivity between functionalized ligands for the nanocompounds and the molecules in the brain (active targeting) [103]. Gold nanocompounds (AuNPs) have been investigated as potential anti‐A*β* therapeutic agents [104]. Hou et al. had created 3.3‐nm L‐ and D‐glutathione which can be utilized as AuNPs. Results showed that these NPs prohibit A*β*42 aggregation and were able to cross the BBB after intravenous administration without considerable toxicity. Furthermore, the functionalization of nanocompounds for a chiral consistency offers promising therapeutic potential for AD [105].

## 11. Liposome Nanoparticles

Liposomes have been widely used as drug carriers, yet their large‐scale production demands organic solvents, particularly for drug entrapment, making scale‐up laborious [106]. The optimized liposomal synthesis exhibited a homogeneous particle size of 102 ± 3.3 nm and a low PI of 0.28 ± 0.03, indicating a minimal vesicle size having the capability for efficient drug encapsulation. In addition, a high encapsulation adequacy (EE) of 84.91*%* ± 3.31*%* was achieved, which can lead to enhanced drug bioavailability. Also, in vitro drug release studies demonstrated a controlled release profile, enhancing assimilation of DNP via the nasal route [107]. Researchers have shown that curcumin‐loaded liposomes have been able to deliver drugs to the CNS through the BBB, displaying anti‐Alzheimer′s effects [108]. Scientists developed a curcumin liposomal formularization with mucoadhesive features of intranasal delivery. In vivo studies demonstrated improved brain bioavailability following intranasal administration, indicating that xanthan gum‐covered curcumin liposomes represent an innovative and efficacious drug delivery system specifically engineered for brain targeting via the nasal route [109].

## 12. Clinical Studies for the Treatment of AD

Current therapeutic strategies for AD have been undergoing clinical studies. In a transgenic mouse paradigm of AD, aducanumab has exhibited the capability of crossing the BBB and attach with parenchymal A*β*, thereby reducing both soluble and insoluble A*β* levels in a dose‐dependent manner. This reduction has been observed to be correlated to a deceleration of disease progression, which has been evaluated by the Clinical Dementia Rating—Sum of Boxes and the Mini‐Mental State Examination scores. Should the outstanding Phase 3 clinical trials illustrate a rebate in clinical slope, this would lead to upholding the hypothesis of amyloid [110].

At present, CREAD clinical trials converge at investigating compounds aimed at enhancing efficacy, safety, and benignity as paralleled to a placebo in participants with prodromal to moderate AD. In addition, elenbecestat, a BACE‐1 inhibitor, is another therapeutic agent being investigated. A Phase 2b trial of elenbecestat in amyloid‐PET‐positive patients with mild cognitive impairment (MCI), prodromal AD, or mild AD displayed a dose‐dependent reduction in CSF A*β* levels; however, there were no significant improvements in the AD Composite Score or the Clinical Dementia Rating—Sum of Boxes (CDR‐SB) score [111].

## 13. Conclusion

In this study, the information and past research that deal with AD, drug delivery system, and metal nanocompounds have been comprehensively covered. The various causes of AD have also been discussed because of the importance of understanding this disease and its prevalence in modern lifestyles.

## Funding

No funding was received for this manuscript.

## Conflicts of Interest

The authors declare no conflicts of interest.

## Data Availability

The data that support the findings of this study are available on request from the corresponding author. The data are not publicly available due to privacy or ethical restrictions.
